# Inter-Allelic Prion Propagation Reveals Conformational Relationships among a Multitude of [*PSI*] Strains

**DOI:** 10.1371/journal.pgen.1002297

**Published:** 2011-09-29

**Authors:** Jia-Yu Lin, Tzu-Ya Liao, Han-Chung Lee, Chih-Yen King

**Affiliations:** 1 Institute of Molecular Biology, Academia Sinica, Taipei, Taiwan; 2 Graduate Institute of Life Sciences, National Defense Medical Center, Taipei, Taiwan; Brown University, United States of America

## Abstract

Immense diversity of prion strains is observed, but its underlying mechanism is less clear. Three [*PSI*] prion strains—named VH, VK, and VL—were previously isolated in the wild-type yeast genetic background. Here we report the generation and characterization of eight new [*PSI*] isolates, obtained by propagating the wild-type strains with Sup35 proteins containing single amino-acid alterations. The VH strain splits into two distinct strains when propagated in each of the three genetic backgrounds, harboring respectively single mutations of N21L, R28P, and Gi47 (*i.e.* insertion of a glycine residue at position 47) on the Sup35 N-terminal prion-forming segment. The six new strains exhibit complex inter-conversion patterns, and one of them continuously mutates into another. However, when they are introduced back into the wild-type background, all 6 strains revert to the VH strain. We obtain two more [*PSI*] isolates by propagating VK and VL with the Gi47 and N21L backgrounds, respectively. The two isolates do not transmit to other mutant backgrounds but revert to their parental strains in the wild-type background. Our data indicate that a large number of [*PSI*] strains can be built on three basic Sup35 amyloid structures. It is proposed that the three basic structures differ by chain folding topologies, and sub-strains with the same topology differ in distinct ways by local structural adjustments. This “large number of variations on a small number of basic themes” may also be operative in generating strain diversities in other prion elements. It thus suggests a possible general scheme to classify a multitude of prion strains.

## Author Summary

A prion is a host-encoded infectious protein that can usually adopt many distinct self-propagating conformations, referred to as prion strains. Upon interspecies transmission, a prion isolate can mutate and give rise to novel strains. It is not well understood how the strain diversity is created. Here we use the [*PSI* ^+^] element, the self-propagating conformers of the yeast Sup35 protein, to study the generation of prion strain diversity. We obtain eight new [*PSI*] isolates by propagating three wild-type strains with mutant Sup35 proteins. The inter-conversion relationships among newly derived strains are complex. A single mutant strain often splits into two strains when propagated with another mutant Sup35 sequence. All isolates however revert to their original parental strains in the wild-type background. Thus, although the new prion isolates appear independent, they all maintain some common character of the parents, likely a shared main chain folding pattern, and can therefore be classified together with the parents into just three strain groups. Our results suggest that many seemingly distinct prion strains might be linked, and the number of independent strain groups might be far smaller than the number of all possible prion strains.

## Introduction

Independent prion isolates that exhibit distinct biological characteristics are identified as prion strains. Successful propagation of yeast [*PSI*] prion strains in pure protein solutions unambiguously established that [*PSI*] strains were amyloid conformers of the same Sup35 protein [1]–[3]. Using polymorphic amyloid fiber preparations of the same mouse prion protein (mPrP), strain variations were induced in pure-breed transgenic mouse lines, similarly demonstrating the essential role of protein conformations in determining mammalian prion strain phenotypes [4].

The Sup35 protein is a subunit of the translation termination factor. The incorporation of the soluble Sup35 protein into [*PSI*] aggregates impedes cellular translation termination and hence increases the read-through of nonsense mutations [5], [6]. Three [*PSI*] strains —VH, VK, and VL—were isolated previously [7]. They were distinguished by how the nonsense suppression activity was altered when propagated in cells expressing mutant Sup35 proteins, each bearing a single amino-acid change on the N-terminal prion-forming segment. Electron microscopy and mass per length measurements revealed morphological differences of the three types of prion fibers [8]. Hydrogen exchange measurements by nuclear magnetic resonance (NMR) spectroscopy and proline-scan mutagenesis further indicated that distinct but overlapping segments of the Sup35 protein were used to assemble different [*PSI*] strains [9], [10].

Inter-species and inter-allelic transmission of the mammalian prion frequently result in the appearance of novel disease phenotypes, including changes in incubation period, tissue tropism, and brain lesion distribution [11]–[15]. Experiments on mouse-adapted scrapie strains provided many examples. Bruce and Dickinson [12] classified mouse-adapted strains into three groups based on the stability of their properties on passage in backgrounds with different *Sinc* alleles, which were later demonstrated to be congruent to *Prnp* alleles [16]. Class I strains, such as ME7, 139A, and 22C, could be stably propagated by both *Prnp* ^a^ and *Prnp* ^b^ alleles, encoding proteins differing at two amino-acid positions. Class II strains were stable in the *Prnp* background they were originally isolated but changed their characteristics when propagated by the other allele. For example, the 22A strain was stable in *Prnp^b^* backgrounds but gradually changed to the 22F strain in *Prnp^a^* backgrounds. Class III strains were intrinsically unstable. For instance, the 87A strain was cloned by end-point dilution in a *Prnp^a^* background. It could suddenly change to the ME7 strain in the same background [13]. The great mutability and potential diversity of prion strains was vividly demonstrated recently by selection in cell culture of spontaneously occurred drug-resistant prion isolates from a sensitive parental strain [17]. In the “protein-only” framework, the emergence of new prion strains was attributed to the appearance of novel prion structures. It was thought that new conformers arose from adaptation and selection of structures that were more compatible with the new host environment [15], [18].

Large numbers of possible prion structures require explanation. We previously observed that the VH strain split into two strains when propagated with Sup35(R28P) and Sup35(Gi47) mutants [10]. The splitting of VH was later discovered in the Sup35(N21L) background as well. Here, we characterize the relationships between the “split strains” and extend the analysis further to include experiments with the VK and VL strains. Our goal is to reveal the nature of the new prion isolates in order to achieve better understanding of the mechanisms that generate prion strain diversity.

## Results

### Eight [*PSI*] Isolates

**Figure 1:**
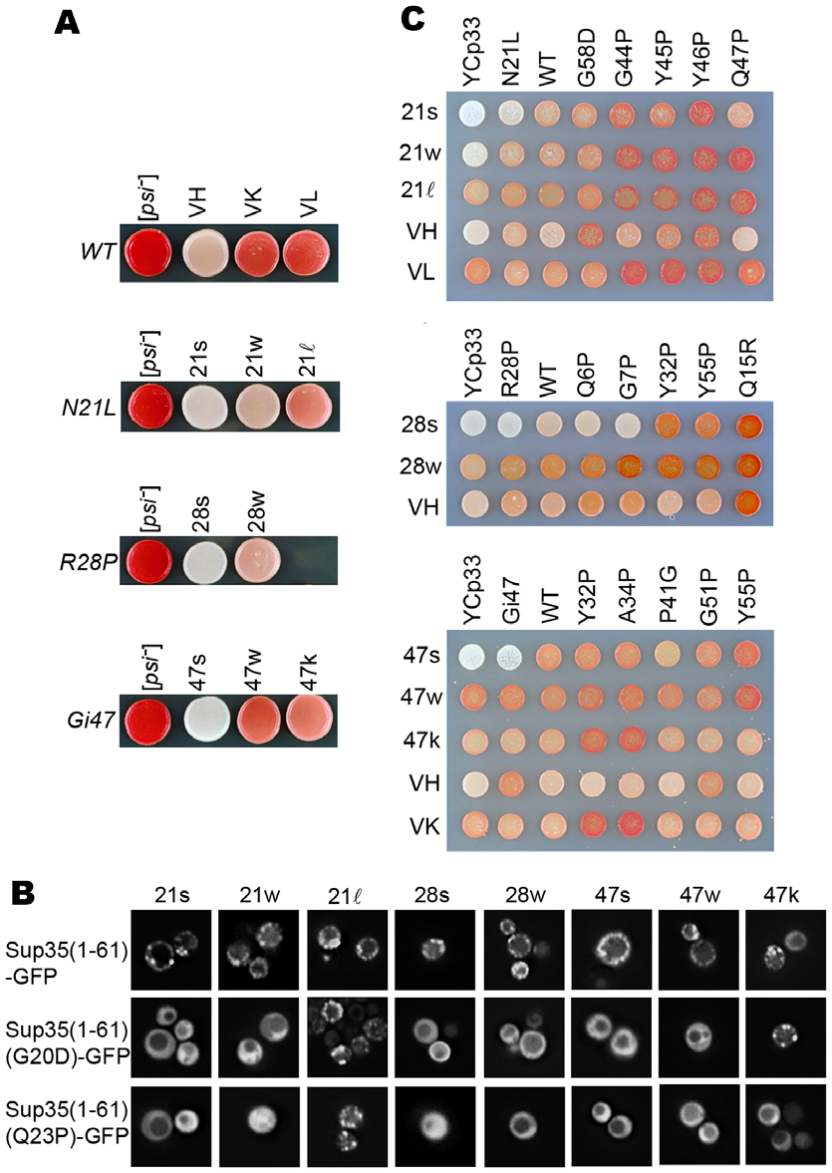
[*PSI*] strains. (A) Collection of [*PSI*] strains. The genetic background is indicated on the left. [*PSI*] strains are indicated on top. (B) Strain typing by GFP labeling. The strain type is indicated on top. GFP fusion constructs are indicated on the left. Three types of labeling are observed: the 47k strain exhibits particulate labeling by Sup(1-61)-GFP and Sup(1-61)(G20D)-GFP, but diffused GFP fluorescence with Sup(1-61)(Q23P)-GFP; the 21ℓ strain exhibits particulate labeling by all three GFP fusion constructs; 21s, 21w, 28s, 28w, 47s and 47w are only labeled by Sup(1-61)-GFP. (C) Strain typing by colony color changes. A set of yeast centromere-based plasmids (YCp33) encoding the wild type Sup35 protein and single mutants (indicated on top of each panel) is introduced into yeast bearing different [*PSI*] strains (indicated on the left). Each [*PSI*] strain gives a distinctive color pattern.

To propagate the three [*PSI*] strains with mutant Sup35 proteins, we first generated heterozygotes by mating [*PSI* ^+^] cells with yeast bearing mutations on the *SUP35* gene. After meiosis, the prion was transmitted to mutant haploid backgrounds. When the VH strain was propagated with Sup35(N21L), two [*PSI*] strains with different nonsense suppression activity were isolated. They were designated 21strong (21s) and 21weak (21w) according to the strength of nonsense suppression (Figure 1A). Four independent heterozygotes were analyzed by random sporulation; the spores showed consistent prion strain distributions (Table 1). Efficient transmission of the VL strain in the mutant background further resulted in a weak strain designated 21ℓ (Figure 1A). The 21ℓ strain could be distinguished from 21w by the darker colony color in the same genetic background (Figure 1A). When Sup(1-61)-GFP—consisting of the first 61 amino-acid residues of Sup35 fused in front of the green fluorescent protein (GFP)—was expressed in the cell, the prion aggregates of all three isolates were visualized by microscopy as numerous fast-moving green particles. The 21ℓ isolate was further distinguished from 21s and 21w by using two additional GFP fusion constructs, Sup(1-61)(G20D)-GFP and Sup(1-61)(Q23D)-GFP [19]; both labeled 21ℓ but did not associate strongly with 21s and 21w (Figure 1B). Therefore three [*PSI*] isolates with distinguishable characteristics were established in the Sup35(N21L) background. Similar experiments were performed with the Sup35(R28P) and the Sup35(Gi47) backgrounds to obtain another 5 new [*PSI*] strains: 28s, 28w, 47s, 47w and 47k (Figure 1A). The first 4 strains were derived from VH by passage in the R28P and Gi47 backgrounds, respectively. The 47k strain was derived by propagating VK in the Gi47 background. It was distinguished from 47s and 47w by colony color differences and by labeling with Sup(1-61)(N20D)-GFP (Figure 1B). Strain competition experiments established the dominance relationships as 21s>21w>21ℓ, 28s>28w, and 47s>47k>47w (Figure S1).

**Table 1:** [*PSI*] strain distribution of random spores.

	28s	28w	VH	[*psi* ^-^](%±SD)
VH x 28[*psi* ^-^]	39.6±1.6	2.9±1.1	51.7±2.9	5.6±1.1
	**47s**	**47w**	**VH**	**[** ***psi*** **^-^]**
VH x 47[*psi* ^-^]	22.2±2.4	5.8±1.4	47.7±1.5	24.1±2.7
	**21s**	**21w+VH**	**[** ***psi*** **^-^]**	
VH x 21[*psi* ^-^]	4.0±0.7	54.3±3.8	41.6±3.9	

n = 4 each. Percentages are averaged directly to obtain the mean and the standard deviation (SD). More than 200 random spores are analyzed for each experimental repeat. The 21w and VH strains are counted together (bottom row) due to their similar colony color. Their coexistence is verified by analyzing randomly selected colonies (see Methods: spore analysis).

### Inter-Conversion Relationships

We investigated the relationships between newly isolated strains by transmitting purified prion particles directly to all genetic backgrounds. Prion particles were labeled with a Sup(1-80)-GFP construct consisting of their respective Sup35 sequence and a C-terminally attached StrepII tag, and then purified by StrepTactin affinity columns [1], [19]. To better distinguish different [*PSI*] strains, we established panels of centromere-based plasmids that expressed full-length Sup35 mutants from the native promoter, each with a single amino-acid substitution on the N-terminal prion-forming domain. The new [*PSI*] strains were then distinguished by characteristic changes in nonsense suppression when the mutant Sup35 proteins were co-expressed (Figure 1C). After particle transformation, prion strain types of all infected (*i.e.* [*PSI* ^+^]) cells were thoroughly and unambiguously determined by colony color, by differential GFP-labeling (described in the previous paragraph) and by observing strain-specific changes of nonsense suppression in response to the co-expression of mutant Sup35. All samples of prion particles had good specificity except 21s, which gave rise to majority of colonies with white and pink sectors in the N21L background (Table 2). The sectors could be streak-purified to form stable colonies of the 21s and 21w strain type, respectively. Sectored colonies persisted even when the infectious material was serially diluted and no single-colored transformants remained (Table 2). We further confirmed that the observed lack of specificity was not an artifact caused by labeling and purification with the Sup(1-80) fragment: particles labeled with the full-length Sup35(N21L) protein similarly gave rise to sectored colonies (Table 2). These data indicated that 21s particles could efficiently nucleate both the 21s and 21w conformations in the cell—the fact that the 21w cells emerged as sectors but not pure colonies when the infectious material was diluted indicated that their appearance was unlikely caused by some contamination. It was thus inferred that in cells harboring the 21s strain, in addition to the true breeding of 21s, 21w conformers were continuously thrown off from the 21s seeds as well. In contrast to the mutable nucleation by 21s, the 21w particles were biologically stable, giving rise to 21w transformants exclusively (Table 3).

**Table 2:** 21s generates 21w.

Particles	Strain type
21s (FL)	10s, 29(s/w)/224
21s (1-80)	3s, 1w, 10(s/w)/224
3x dilution	6(s/w)/224
10x dilution	1s, 3(s/w)/224
20x dilution	2(s/w)/224

21s (FL): 21s particles labeled and purified with the full-length Sup35(N21L)-StrepII.

21s (1-80): 21s particles labeled and purified with Sup(1-80)(N21L)-GFP-StrepII.

“3s, 1w, 10 (s/w)/224”: 3 colonies of the strong strain type, 1 colony of the weak strain type and 10 sectored colonies (strong/weak) are observed out of 224 colonies of yeast spheroplasts which receive a co-transformed YCp111 plasmid.

**Table 3:** Inter-conversion of [*PSI*] strains by particle infection.

Particle→Background	Strain type
21s (1-80) → *N21L*	6s, 4w, 29(s/w)/224
21s (1-80) → *R28P*	10s, 23w, 1(s/w)/224
21s (1-80) → *Gi47*	46s/224
21s (1-80) → *WT*	108VH/223
21w (1-80) → *N21L*	78w/224
21w (1-80) → *R28P*	86w/224
21w (1-80) → *Gi47*	0/224
21w (1-80) → *WT*	23VH/223
21ℓ (FL) → *N21L*	58ℓ/224
21ℓ (FL) → *R28P*	0/224
21ℓ (FL) → *Gi47*	0/224
21ℓ (FL) → *WT*	12VL/218
28s (1-80) → *R28P*	64s, 8w/224
28s (FL) → *R28P*	76s, 3w/224
28s (1-80) → *Gi47*	48s/224
28s (1-80) → *N21L*	32s, 4w, 18(s/w)/224
28s (1-80) → *WT*	96VH/224
28w (1-80) → *R28P*	138w/224
28w (1-80) → *Gi47*	0/224
28w (1-80) → *N21L*	29w/224
28w (1-80) → *WT*	67VH/224
47s (1-80) → *Gi47*	93s/224
47s (1-80) → *R28P*	14s, 66w/224
47s (1-80) → *N21L*	53s, 10w, 22(s/w)/224
47s (1-80) → *WT*	118VH/224
47w (1-80) → *Gi47*	19w/82
47w (1-80) → *R28P*	70w/224
47w (1-80) → *N21L*	1s, 5w, 2(s/w)/224
47w (1-80) → *WT*	48VH/224
47k (1-80) → *Gi47*	135k/224
47k (1-80) → *R28P*	0/224
47k (1-80) → *N21L*	0/224
47k (1-80) → *WT*	102VK/224

Notations are the same as in Table 2.

**Figure 2:**
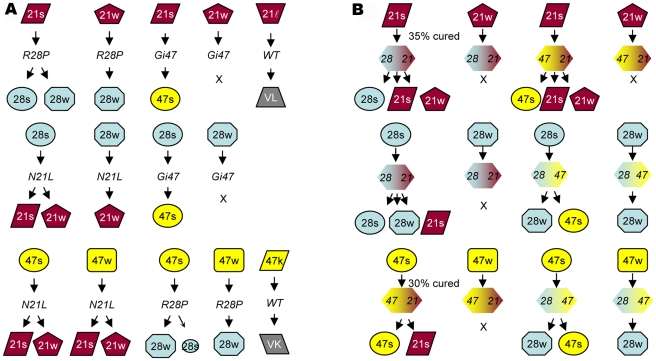
Inter-conversion of new [*PSI*] strains. (A) Infection by prion particles. [*PSI*] strains are represented by geometric shapes with black frames. Genetic backgrounds are in italic. Arrows indicate the direction of transmission and the outcome. “x” indicates [*PSI*] curing. (B) Transmission via mating and sporulation. [*PSI*] strains are transmitted to heterozygous backgrounds (represented by two-colored hexagons) by mating. The heterozygotes are then sporulated. When forming heterozygotes causes [*PSI*] curing of the diploids, the percentage of cured colonies is indicated. 21 =  *N21L*; 28 = *R28P*; 47 = *Gi47*. Prion strains are unambiguously determined by all strain typing methods shown in Figure 1 (see also Table 3 and Table 4).

Cross-background transmissions revealed that two prion isolates from different Sup35 backgrounds did not convert/revert to each other in a direct, simple fashion except the following three cases: (I) when 21ℓ was transmitted to the wild-type background it reverted to VL; (II) 47k reverted to VK in the wild-type background; (III) 21w converted to 28w in the R28P background, and 28w reverted back to 21w in the N21L background (Table 3, data summarized in Figure 2A). The weak strain derived in the R28P background by 21w infection and vice versa, were judged identical to the original 28w and 21w strains, respectively, by our stringent strain-typing protocols described above. Another weak strain, 47w, could convert to 28w but the opposite transmission (28w→47) caused [*PSI*] curing; the 47w strain converted to a mixture of 21s and 21w in the N21L background with lower efficiency, but 21w could not be converted to 47w. Cross-transmission with strong strains (21s, 28s, and 47s) yielded more complex patterns: transmission of 28s and 47s to the N21L backgound generated 21s, sectored colonies (21s/w), and 21w (listed according to frequency, in descending order); transmission of 21s and 47s to the R28P background generated majorly the 28w strain and to a lesser extent, the 28s strain; transmission of 21s and 28s to the Gi47 background generated 47s only (Table 3, Figure 2A). In summary, cross-transmission of weak (w)-strains generally resulted in the appearance of w-strains (except 47w); cross-transmission of strong (s)-strains resulted in the appearance of both strong (s) and weak (w) strains; and the 21ℓ and 47k strains were never produced from transmission of s- and w-strains. The 21s, 21w, 28s, 28w, 47s, and 47w strains all converted to the VH strain in the wild-type background (Table 3).

**Table 4:** Inter-conversion of [*PSI*] strains: transmission by mating and sporulation.

	47s	47w	28w	[*psi* ^-^](%±SD)	
28s x 47[*psi* ^-^]	36.2±5.4	0	37.9±5.9	25.8±3.9	
28w x 47[*psi* ^-^]	0	0	22.6±9.4	77.3±9.4	
47s x 28[*psi* ^-^]	43.6±6.2	0	27.5±4.6	28.7±3.6	
47w x 28[*psi* ^-^]	0	0	34.2±3.6	65.7±3.6	
	**21s**	**21w**	**47s**	**[** ***psi*** **^-^]**	
21s x 47[*psi* ^-^]	15.2±3.5	4.6±1.1	28.2±5.3	51.8±7.0	
21w x 47[*psi* ^-^]	0	0	0	100	
47s x 21[*psi* ^-^]	36.3±2.1	0	32.6±2.3	31.0±2.1	
47w x 21[*psi* ^-^]	0	0	0	100	
	**28s**	**28w**	**21s**	**21w**	**[** ***psi*** **^-^]**
28s x 21[*psi* ^-^]	36.4±7.0	9.9±4.9	43.2±4.7	0	10.4±3.5
28w x 21[*psi* ^-^]	0	0	0	0	100
21s x 28[*psi* ^-^]	42.9±1.9	0	33.7±3.6	6.2±1.9	17.0±3.4
21w x 28[*psi* ^-^]	0	0	0	0	100
	**28s**	**28w**	**[** ***psi*** **^-^]**		
28s x 28[*psi* ^-^]	94.2±2.2	2.1±1.2	3.5±1.1		
28w x 28[*psi* ^-^]	0	64.1±2.8	35.8±2.8		
	**47s**	**47w**	**[** ***psi*** **^-^]**		
47s x 47[*psi* ^-^]	98.1±1.1	0	1.8±1.1		
47w x 47[*psi* ^-^]	2.3±0.5	75.0±6.1	22.5±5.8		
	**21s**	**21w**	**[** ***psi*** **^-^]**		
21s x 21[*psi* ^-^]	81.5±3.0	0	18.4±3.0		
21w x 21[*psi* ^-^]	0	73.4±4.5	26.5±4.5		
	**VH**				
VH x wt[*psi* ^-^]	100				

n = 6-8 each. For a single experimental repeat, >200 spores are analyzed (see Methods: spore analysis).

### VH Is a Single Strain

**Figure 3:**
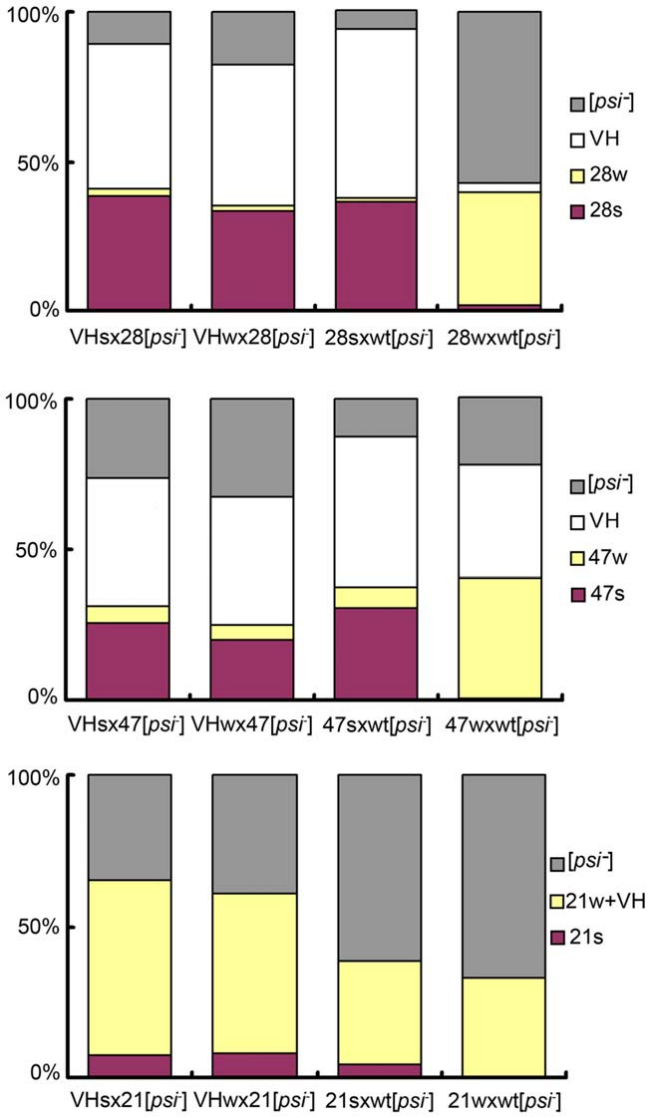
VH is a single strain. Bar graphs of prion strain type distribution of yeast spores. Top panel: spores from the *wt/R28P* background; middle: *wt/Gi47* background; bottom panel: *wt/N21L* background. Genetic cross is indicated under each bar. Prion strain types are color-coded. Each bar represents the average of 4 distributions, each obtained from an independent heterozygote. For each heterozygote, more than 200 random spores are counted (see Methods: spore analysis for experimental procedures, and Table S1 for exact mean values and standard deviations). The heterozygotes formed by VH_s_ and VH_w_ give similar spore distribution, suggesting that VH_s_  = VH_w._

We asked if the original VH strain contained two unresolved sub-strains, which respectively gave rise to the s- and w- strains in the mutant backgrounds. Two types of diploids were obtained when 28s and 28w cells were crossed with a [*psi* ^-^] wild-type isolate. The former cross generated diploids with stronger nonsense suppression activity; after meiosis, they produced random spores of mainly the 28s type (for R28P spores), and the VH type (for wild-type spores, designated VH_s_) (Figure 3, Table S1). Most cells from the latter cross exhibited weak nonsense suppression and produced spores of mainly the 28w type, and the VH type (designated VH_w_) (Figure 3, Table S1). These data were reproduced from 4 independent heterozygotes for each type of genetic cross. We tested whether VH_s_ and VH_w_ remembered their provenance. When the VH_s_ and VH_w_ spores were each back-crossed with a [*psi* ^-^] R28P isolate, the respective diploids exhibited identical nonsense suppression activity; both generated VH spores and predominantly 28s spores, suggesting that VH_s_ was the same as VH_w_— there was no memory (Figure 3, Table S1). Similar results were obtained from experiments of crossing the wild-type [*psi* ^-^] isolate with the 21s and 21w strains, as well as with the 47s and 47w strains (Figure 3, Table S1). Thus there is no evidence to indicate that VH is composed of two undistinguished prion strains. This does not rule out, however, that the VH strain is composed of an ensemble of short-lived dynamic structures (compared with host’s generation time), and that they entropically contribute to the robust propagation observed for VH [20]. Such a possibility, if confirmed, would suggest that VH exists as a quasispecies, as proposed by Li *et al*. for a mammalian prion strain [17], [21]-[23].

### Two Distinct [*PSI*] States in Every Heterozygote Type

We were always able to identify at least two [*PSI* ^+^] states in heterozygous diploid backgrounds. For example, consider the N21L/R28P background: When we crossed 21s with 28[*psi* ^-^] (*i.e.* the [*psi* ^-^] derivative of 28s), about 65% of the diploids remained [*PSI* ^+^], which were stable and gave random spores of the 21s, 21w and 28s type (Table 4, data summarized in Figure 2B). The cross in the opposite direction (28s X 21[*psi* ^-^]) resulted in 100% [*PSI* ^+^] diploids, which in turn gave 21s, 28s, and 28w spores. These data were reproduced from 6-8 independent heterozygotes for each type of genetic cross. The fact that the same genetic background contained two distinguishable prion states, imparted by 21s and 28s, respectively, further supported the aforementioned conclusion that the 21s and the 28s strain were not equivalent. Similar results were obtained from heterozygotes consisting of other combinations of *SUP35* alleles: there were two different [*PSI* ^+^] states in the R28P/Gi47 background; one (derived from 28s X 47[*psi* ^-^] and 47s X 28[*psi* ^-^]) gave rise to 28w and 47s spores and the other (derived from 28w X 47[*psi* ^-^] and 47w X 28[*psi* ^-^]) gave rise to 28w spores only. And the two [*PSI* ^+^] states in the N21L/Gi47 background gave rise to spores of 47s, 21s, and 21w type (from 21s X 47[*psi* ^-^]) and spores of 47s and 21s type (from 47s X 21[*psi* ^-^]), respectively (Table 4, Figure 2B). Furthermore, by comparison of Figure 2B with Figure 2A, it was clear that inter-background prion transmission via an intermediate heterozygote stage restricted ensuing prion strain types. For example, direct transmission of 21s prion particles to the R28P background resulted in 28s and 28w, but only 28s was derived from 21s/28 heterozygotes (together with 21s and 21w). Although the absence of 28w spores seemed consistent with 28w curing in the N21L/R28P background (28w X 21[*psi* ^-^], Figure 2B, the second row), [*PSI*] curing mechanism could not explain why 21w spores were obtained— by a similar argument, 21w should have been cured in the presence of R28P allele (21w X 28[*psi* ^-^], Figure 2B, the first row) but they didn’t. Further investigation is required to fully understand the heterozygous [*PSI*] structures and the molecular mechanism for strain-type restriction.

### [*PSI*] Strain Groups

Taken together all results described above, we arrive at the following group structure:

{21s, 28s, 47s, 47w, VH, (21w, 28w)}; {47k, VK}; {21ℓ, VL}

A strain group is defined as a collection of prion strains whose propagation with a group member’s *SUP35* allele gives rise to strains in the same group. While 21w and 28w are revertible, transmission through the wild type Sup35 and back nevertheless generate s-strains; VK and VL form their own independent groups, respectively.

### Structural Incompatibility of 21w and 28w Fibers

**Figure 4:**
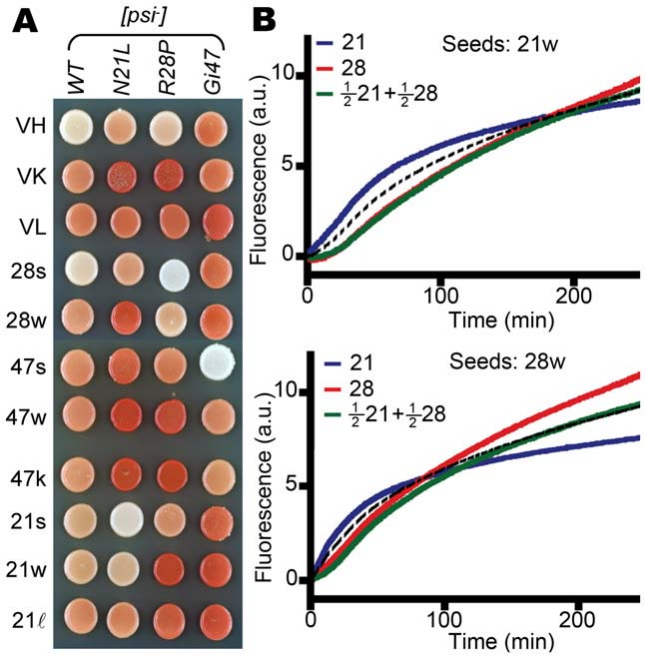
Distinct structures of [*PSI*] strains. (A) Yeast harboring a [*PSI*] strain (labeled on the left) is mated with different genetic backgrounds (labeled on top). Homozygotes always exhibit stronger suppression of the *ade2-1* nonsense mutation, resulting in lighter colony color. (B) Nucleated growth of prion fibers monitored by ThT fluorescence *in vitro*. Top panel: 21w seeds. Bottom: 28w seeds. 21 =  1 µM solution of Sup(1-253)(N21L) (blue); 28 =  1 µM solution of Sup(1-253)(R28P) (red). The 0.5∶0.5 mixture (green) exhibits lower ThT fluorescence at early time points compared with the averaged signal (dotted black line) of the two pure solutions.

We next demonstrated the structural difference of prion fibers of different mutants. As mentioned earlier, although the 21w strain was converted to the 28w strain in the R28P background and vice versa, the 21w strain and the 28w strain were nevertheless cured in the heterozygous N21L/R28P background (Figure 2B). In fact, for all prion strains and *SUP35* alleles investigated here, the nonsense suppression activity of a strain was always weakened when mated with cells expressing different Sup35 proteins (Figure 4A). We compared the growth kinetics of amyloid fibers nucleated from solutions of Sup(1-253)(N21L), Sup(1-253)(R28P), and equal molar mixture of both by thioflavin (ThT) fluorescence [24]. The presence of two proteins in the same solution resulted in slower ThT fluorescence increase at early time points, compared with averaged fluorescence signals of the two pure solutions, regardless whether prion particles of the 21w or the 28w strain type were used as seeds (Figure 4B). These results suggested that the different Sup35 proteins were not compatible in adopting the same structures of either 21w or 28w; since if they had been, the seeding curve of the mixed solution should have followed closely to the curves of the two pure solutions averaged. The growth interference of 21w and 28w in the N21L/R28P background could be viewed as an extreme case of overdominance, also observed for several mammalian prion strains, of which incubation periods were longer in F1 *Prnp* heterozygotes than in either of the homozygous parents [11], [14].

## Discussion

The inter-conversion of the new yeast prion strains bears striking resemblance to that of mammalian strains. We describe 4 parallels: First, the VH strain gives rise to 2 distinct strains in each of the mutant backgrounds. This is similar to the generation of the drowsy (DY) and hyper (HY) prion strains in golden hamsters by infection with a cloned mink strain [25]. Second, transient propagation of the 28s strain in the Gi47 background generates the 28w strain (28s→47s→28w). This is analogous to the generation of a new mouse prion strain by passing the 139A strain through golden hamsters [26]. Third, the 21s strain is unstable which continuously generates the 21w strain; the mouse-adapted scrapie strain 87A, is similarly unstable, continuously generating the ME7 strain [12]–[4]. Fourth, the 47s and 47w strains are stable in their native background, but each gives rise to sectored colonies in the N21L background. Similarly, elk infected with the chronic wasting disease (CWD) can harbor either of the two stable prion strains— CWD1 or CWD2, but each strain gives rise to a CWD1/CWD2 mixture when propagated with a deer sequence, which differs from the elk protein by a single amino-acid change [27]. These parallels, adding to an already impressive array of analogies [7], [28]–[31], argue strongly that the same molecular mechanism is at play for the generation of prion strain diversities.

We demonstrated that the VH strain was a single strain. This finding indicated that the conversion of VH into two separate strains in a mutant background was not due to strain selection from a doubly infected host but rather an adaptation process resulting in two different structures. The adaptation process could at best involve selection and evolution of dynamic microstates [15], [17].

Most of the new strains derived from VH give rise to two strains when propagated by a different Sup35 sequence. Yet they all revert to the VH strain in the wild-type background. Thus it is best to describe the six strains collectively as a strain group. We further demonstrate that the VK and VL strains could also form their own groups. The concept of strain group may be useful to better relate a large number of prion strains. For example, three stable [*PSI*] strains—47s, 47w, and 47k—can be induced in the Sup35(Gi47) background. The closer relation between 47s and 47w (as compared with 47k) cannot be assumed a priori, but is revealed by observing their convergence to the VH strain in the wild-type background. It is possible that there are other strain groups and there might be mutant Sup35 sequences that form a group which excludes wild-type strains.

What could be the chemical basis of strain groups? The VH, VK, and VL strains, archetype members of the 3 [*PSI*] groups, involve distinct but overlapping stretches of the Sup35 polypeptide to form cross-β amyloid structures [9], [10]. This suggests that each strain group has a characteristic chain folding topology which cannot easily inter-convert. Sub-strains would then differ by local structural adjustments to maintain compact side chain packing inside prion fibers [32], [33], or involve accessory sequence elements [34]. To support this idea, we note that the *SUP35(N27P)* allele is compatible with the VH and the VL strain but cures VK [10]. Co-expression of the mutant protein in wild-type cells harboring the VK strain always results in [*PSI*] curing instead of strain-type conversion to VH or VL [10]. Inter-group conversions can however take place when the protein being nucleated contains alterations of a large sequence segment, as freeing parts of the polypeptide from interactions which hold them in place, can allow their reorganization to adopt alternative chain-folding patterns. Examples of such conversion include the generation of the VK strain by propagating VL with a truncated Sup(1-40) construct [10], and the generation of the [*PSI* ^+(SCS)^] strain by cross-species propagation with the Sup35 protein from *Candida albicans* [35]. Cross seeding of more refractory sequences could also induce different prion strains, but this usually happens with efficiencies orders of magnitude lower. Particular examples include the induction of [*PSI*] from [*PIN*] [36], and probably, the induction of [*CHI* ^+^_PM_] strains from [*PSI*] [37].

It is important to test if strain groups can be further applied to catalogue all prion strains across species in a simple and consistent manner. A good place to start is to attempt classification of all [*PSI*] strains of *Saccharomyces sensu stricto* species, employing ideas developed by Chen *et al*. [38]–[40]. In addition, the concept of strain groups is likely applicable to the mammalian prion. It would be useful to investigate whether all regional field prion isolates of a mammalian species, such as human sporadic Creutzfeldt-Jakob disease (sCJD) strains or sheep scrapie strains, belong to the same strain group. If so, to understand why a strain group occurs more frequently than others might lead to insight regarding etiologies or cellular environments that are conducive for the formation and propagation of a specific prion chain folding topology. Finally, it is interesting that many strains in the VH group come in pairs. Could there also be a VH twin which escapes detection because it is toxic to the host [41] or it is too weak to propagate?

## Methods

### Yeast Strains and General Methods

Experiments were performed with the 5V-H19 genetic background (*SUQ5 ade2-1(UAA) can1-100 leu2-3,112 ura3-52*) [42]. gα5V-H19-*Prp*Δ*SF* is a *MAT*α, [*psi^-^*], [*PIN^+^*] derivative of 5V-H19, of which the Sup35 (5-55) coding sequence is replaced with the mouse Prp^a^ (94-230) sequence. Colonies of gα5V-H19-*Prp*Δ*SF* are white. Standard protocols were used for media preparation and yeast genetic manipulation [43]. PCR primers are listed in Table S2.

### Mutant Backgrounds

YIp-I-SUPN [7] containing the 1243-bp *SUP35* promoter following by a *Bam*HI restriction site and then the first 114 codons of *SUP35*, was subject to site-directed mutagenesis, using the QuickChange II kit (Stratagene, La Jolla, CA), to generate N21L, R28P, and Gi47 mutations. Mutant plasmids were used as templates for PCR amplification of *SUP35* sequences, using primers CYK-43 and CYK-2. The PCR products were co-transformed with YCplac111 [44] into gα5V-H19-*Prp*Δ*SF* to select red colonies on SC-LEU agar plates. For each mutation, two independent red colonies were isolated and verified by *Bam*HI digestion of genomic PCR products (primers CYK-43 and CYK-15), and by sequencing the first 372 bp of the *SUP35* coding sequence.

### New [*PSI*] Strains

[*PIN* ^+^] [*psi* ^-^] cells with N21L, R28P, or Gi47 mutations were mated with the wild-type 5VH19 derivative (Δ*his4*:*KanMX* [*YCplac111*(*LEU2*)]) bearing prion strains VH, VK, or VL. The diploids were selected and purified by complementation of nutritional markers and then sporulated in bulk (*i.e.* random sporulation). For each [*PSI*] strain-mutant background combination, 4 independent (clonally unrelated) heterozygote colonies derived from 2 genetic crosses (using independent haploids) were isolated. They were subject to spore analysis (see below) to isolate new [*PSI*] strains.

### Spore Analysis

For each individual colony, more than 200 random spores were obtained. They were grouped according to colony color (Figure 1A). To confirm that spores of different colony colors harbored distinct [*PSI*] strain types, 8 spores from each color group were randomly selected for further genotype and strain type analysis. Spores of different *SUP35* genotypes were distinguished from each other by sequencing a colony PCR fragment (primers CYK-43 and CYK-15). Prion strains were distinguished by differential GFP-labeling with Sup(1-61)-GFP, Sup(1-61)(G20D)-GFP, and Sup(1-61)(Q23P)-GFP [19], and by the characteristic changes in nonsense suppression when a panel of plasmids carrying mutant *SUP35* alleles was co-expressed. For the N21L background, the panel of plasmids consisted of YCp33, YCp33-I-SUPF [7], YCp33-I-SUPF(N21L), (G58D), (G44P), (Y45P), (Y46P), and (Q47P); for the R28P background: YCp33, YCp33-I-SUPF, YCp33-I-SUPF(R28P), (Q6P), (G7P), (Q15R), (Y32P), and (Y55P); for the Gi47 background: YCp33, YCp33-I-SUPF, YCp33-I-SUPF(Gi47), (Y32P), (A34P), (P41G), (G51P), and (Y55P) (Figure 1C). The VH and the 21w prion strains could not be easily distinguished by colony color alone, but they had different genotype and could be unambiguously distinguished by characteristic colony color changes in response to the coexpression of the mutant *SUP35* alleles. Consistent results were obtained from independent colonies.

### Strain Competition

Cells harboring different prion strains were transformed with YCp33 (*URA3*) and YCp111 (*LEU2*) [44] respectively and then mated. Diploids were selected and purified on SC-LEU, URA, grown on rich media to lose YCp33 (to allow *URA3* selection again), transformed with strain typing plasmids carrying a mutant *SUP35* allele (described in spore analysis, all with a *URA3* marker), and then strain-typed by colony color changes. The prion strain type of the diploids was also consistently distinguished by strain-specific GFP labeling.

### Prion Particles

[*PSI^+^*] cells were transformed with YEp195-CUP1-SUP(1-80)-GFP-Strep(II)-T [1] or YEp195-CUP1-SUP35(full length)- Strep(II)-T. Prion particles were isolated from the yeast cells and purified by StrepTactin affinity chromatography as described [1], [19], [45]. The Sup35 sequence of the labeling plasmid matched that of the chromosome for each genetic background.

### Prion Infection

Spheroplasts ([*pin* ^-^] [*psi* ^-^]) were prepared as described [1], [19]. Purified yeast prion particles (10 µl in Buffer E (100 mM Tris-HCl, 1mM EDTA, 2.5 mM desthiobiotin, pH 8.0), 0.3-1 µM monomer concentration) and 5 µg YCp111 plasmid (1 mg/ml) were added to 50 µl spheroplasts (10^7^cells/ml) suspended in buffer Z1 (1.2M sorbitol, 10mM Tris-HCl (pH 7.5), 30 mM CaCl_2_) to incubate at 22°C for 15 minutes. One milliliter of buffer Z2 (20% (w/v) polyethylene glycol 3350, 10 mM Tris-HCl (pH 7.5), 30 mM CaCl_2_) was then added to incubate for another 15 min. Spheroplasts were then collected by centrifugation at 2100g (5 min), resuspended in 150 µl Z3 (1M sorbitol, 30 mM CaCl_2_, 1/3 strength YPAD, 33 mg/l leucine, 7 mg/l uracil, 7 mg/l histidine HCl, 7 mg/l tryptophan), and incubated at 30°C for 30 min. The spheroplasts were then plated in top agar (SC-LEU, 1.2M sorbitol, 1.6% (g/ml) Bacto agar) and grew for 3 days at 30°C. LEU^+^ transformants were picked from top agar and transferred to the surface of SC-LEU plates to develop colony color for further analysis of [*PSI*] status by all the strain typing methods described in spore analysis.

### [*PSI* ^+^] Heterozygotes

Haploids of opposite mating types were transformed with YCp33 (*URA3*) and YCp111 (*LEU2*) [44] respectively and then mated. Heterozygotes were selected and purified on SC-LEU, URA. For each heterozygote type described in this work, 4-8 independent (clonally unrelated) colonies derived from 2-4 genetic crosses (using independent haploids) were isolated. They were then subject to spore analysis described above.

### Recombinant Proteins

The plasmid pHis-Sup(1-253), expressing the first 253 amino-acid residues of Sup35 with an N-terminal His_5_ purification tag (sequence MGS_2_H_5_S_2_G_2_), was constructed by replacing the 942-bp *BamHI*/*EcoRI* fragment of pHis-N1-GFP-Strep(II) [1] with a 767-bp *BamHI*/*EcoRI* DNA fragment derived from PCR using CYK-1 and CYK-16 as primers and YCp-I-SupF as the template. The plasmids pHis-Sup(1-253)(N21L) and pHis-Sup(1-253)(R28P), expressing N21L and R28P mutants, respectively, were constructed by replacing the 170 bp wild-type *BamHI*/*BstXI* fragment with those of the mutations, removed from YIp-I-SUPN(N21L) and YIp-I-SUPN(R28P), respectively. The *E. coli* strain BLR (DE3)/pLysS was used to express Sup(1-253)(N21L) and Sup(1-253)(R28P) [46]. Proteins were purified from *E. coli* extracts by Ni-NTA affinity columns (Qiagen) in buffers containing 6M guanidine hydrochloride, which were then removed by reverse phase HPLC using a C8 column as described [47]. They were then lyophilized and stored at −80°C.

### Seeding Assay

Lyophilized Sup(1-253) proteins were dissolved in 8M urea to a final concentration of 100 µM (determined by UV absorption, molar extinction coefficient ε_280_  =  25600, [48]) right before experiments, and then diluted 100 fold to make 2 ml reaction solutions containing 50 mM TrisHCl (pH 8.0), 12.5 µM thioflavin T (ThT), 25 mM glycine, and 0.5 mM EDTA. Fifteen microliters of [*PSI*] particles (∼0.5 µM monomer concentration, labeled with Sup(1-80)-GFP-Strep(II)) were added to the reactions, mixed by gentle rotation of cuvettes, and placed immediately in a Varian Cary Eclipse fluorescence spectrophotometer to start measurement at 25°C without further agitation. ThT fluorescence was measured at 485 nm with excitation at 442 nm (10 nm window width) and 1s averaging time. Seeds alone in reaction buffer were measured and used as blanks. Solutions mock-seeded with only the buffer were used as controls to rule out *de novo* fiber formation.

## Supporting Information

Figure S1 Strain competition. Two haploid colonies bearing different [*PSI*] strains are mated to form diploids, whose strain type is subsequently determined by (A) colony color, (B) characteristic colony color changes in response to the co-expression of mutant Sup35, and (C) strain-specific GFP labeling. Competing prion strains are labeled on the side in panels A and B, and on top in panel C. Strain-typing plasmids are labeled on top in panel B and on the left in panel C.
(TIF)

Table S1 VH is a single strain: prion strain distribution of random spores.
(DOC)

Table S2 Oligonucleotide sequences.
(DOC)
