# Supplementary figures and images for: Inter-Allelic Prion Propagation Reveals Conformational Relationships among a Multitude of [PSI] Strains
Source: PLoS Genet. 2011 Sep 29;7(9):e1002297. doi: 10.1371/journal.pgen.1002297 (PMC3183073; doi:10.1371/journal.pgen.1002297)

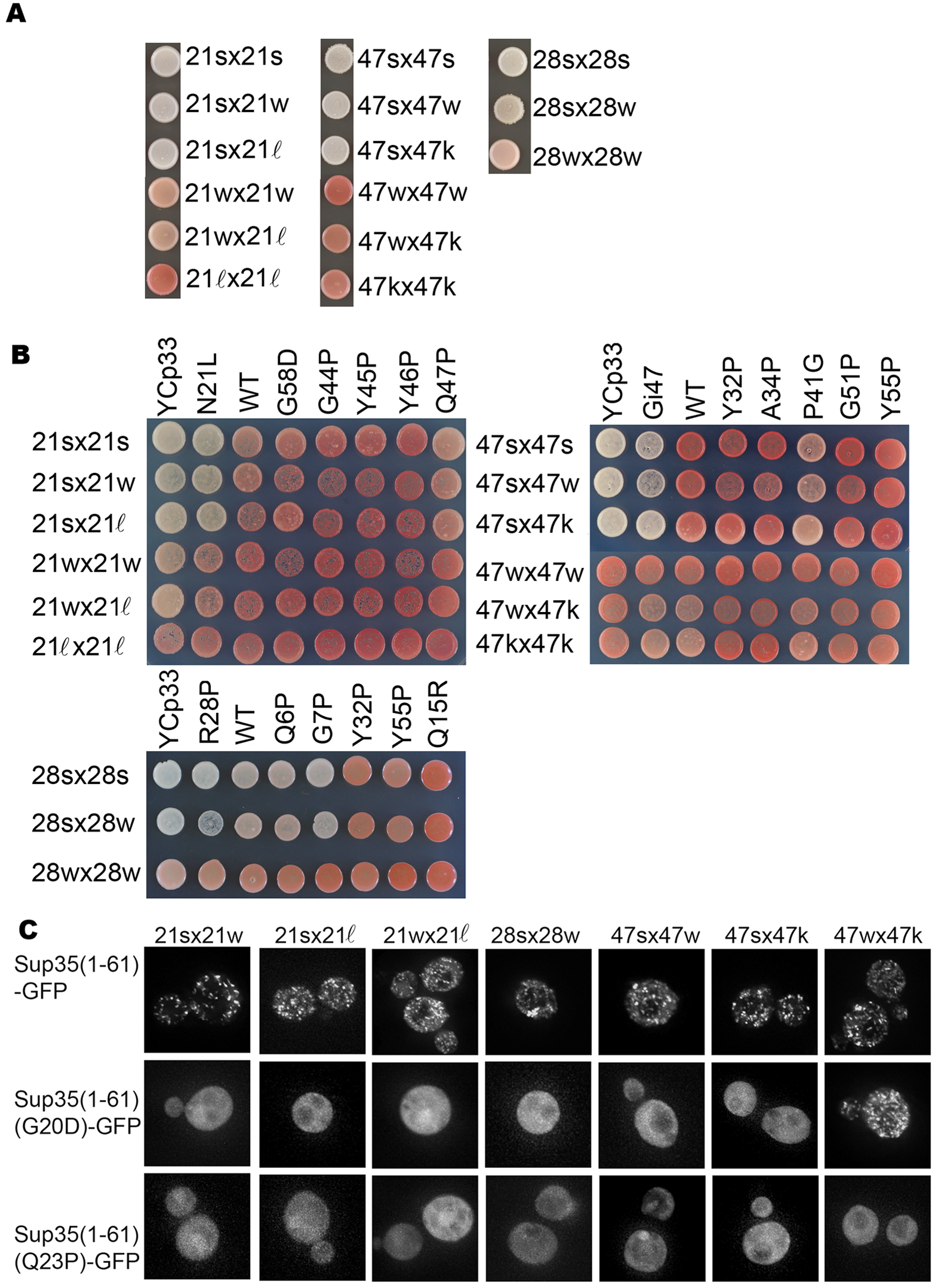

Supplement: Figure S1 — Strain competition. Two haploid colonies bearing different [PSI] strains are mated to form diploids, whose strain type is subsequently determined by (A) colony color, (B) characteristic colony color changes in response to the co-expression of mutant Sup35, and (C) strain-specific GFP labeling. Competing prion strains are labeled on the side in panels A and B, and on top in panel C. Strain-typing plasmids are labeled on top in panel B and on the left in panel C. (TIF) [file pgen.1002297.s001.tif]
