# Supplementary material for: Inter-Allelic Prion Propagation Reveals Conformational Relationships among a Multitude of [PSI] Strains
Source: PLoS Genet. 2011 Sep 29;7(9):e1002297. doi: 10.1371/journal.pgen.1002297 (PMC3183073; doi:10.1371/journal.pgen.1002297)
Supplement: Table S1 — VH is a single strain: prion strain distribution of random spores. (DOC) [file pgen.1002297.s002.doc]

**Table S1. VH is a single strain: prion strain distribution of random spores**

|  | **28s** | **28w** | **VH** | **[*psi*^-^](%±SD)** |
| --- | --- | --- | --- | --- |
| VHs x 28[*psi*^-^] | 38.6±3.3 | 2.5±0.9 | 47.8±2.1 | 10.9±1.0 |
| VHw x 28[*psi*^-^] | 33.0±1.2 | 2.0±0.5 | 47.4±2.0 | 17.5±1.2 |
| 28s x wt[*psi*^-^] | 36.3±3.4 | 0.6±0.1 | 56.6±2.8 | 6.4±2.1 |
| 28w x wt[*psi*^-^] | 2.1±3.3 | 37.5±2.3 | 3.3±2.2 | 56.9±7.4 |
|  | **47s** | **47w** | **VH** | **[*psi*^-^]** |
| VHs x 47[*psi*^-^] | 25.6±2.1 | 5.6±1.3 | 42.2±0.9 | 26.5±1.5 |
| VHw x 47[*psi*^-^] | 19.7±1.3 | 5.2±1.7 | 42.2±3.4 | 32.8±3.0 |
| 47s x wt[*psi*^-^] | 30.7±4.7 | 6.6±2.6 | 50.4±6.5 | 12.2±6.1 |
| 47w x wt[*psi*^-^] | 0.5±0.7 | 39.7±1.5 | 37.2±3.0 | 22.3±2.3 |
|  | **21s** | **21w+VH** | **[*psi*^-^]** |  |
| VHs x 21[*psi*^-^] | 7.2±2.1 | 57.8±5.2 | 34.9±6.3 |  |
| VHw x 21[*psi*^-^] | 8.2±1.1 | 52.5±8.7 | 39.1±9.0 |  |
| 21s x wt[*psi*^-^] | 4.4±3.4 | 34.1±5.2 | 61.3±7.1 |  |
| 21w x wt[*psi*^-^] | 0 | 33.2±6.0 | 66.7±6.4 |  |

n=4 each. Percentages are averaged directly to obtain the mean and the standard deviation (SD). For a single experimental repeat, >200 spores are analyzed (see **Methods**: **spore analysis**).
