# Supplementary material for: Inter-Allelic Prion Propagation Reveals Conformational Relationships among a Multitude of [PSI] Strains
Source: PLoS Genet. 2011 Sep 29;7(9):e1002297. doi: 10.1371/journal.pgen.1002297 (PMC3183073; doi:10.1371/journal.pgen.1002297)
Supplement: Table S2 — Oligonucleotide sequences. (DOC) [file pgen.1002297.s003.doc]

**Table S2.** Oligonucleotide sequences

| CYK-2 | 5’-GGTTGGAATTCCTATTGATATCCTTGCAAATTGTTATTGTAG |
| --- | --- |
| CYK-15 | 5’-GGTTGGAATTCTTAACCTTGAGACTGTGGTTGGAAACC |
| CYK-16 | 5’-GGTTGGAATTCTTAATCGTTAACAACTTCGTCATCCAC |
| CYK-43 | 5’-GTCATCTAGACATATGAAGGCACTGAACCAG |
